# A Review of Methodologies for the Detection, Quantitation, and Localization of Free Cysteine in Recombinant Proteins: A Focus on Therapeutic Monoclonal Antibodies

**DOI:** 10.3389/fmolb.2022.886417

**Published:** 2022-06-27

**Authors:** Clive Metcalfe

**Affiliations:** Division of Biotherapeutics, National Institute for Biological Standards and Control, Potters Bar, United Kingtom

**Keywords:** therapeutic monoclonal antibodies, disulfide bond, free cysteine, quantitative mass spectrometry, and post-translation modification

## Abstract

Free-cysteine residues in recombinant biotherapeutics such as monoclonal antibodies can arise from incorrect cellular processing of disulfide bonds during synthesis or by reduction of disulfide bonds during the harvest and purification stage of manufacture. Free cysteines can affect potency, induce aggregation, and decrease the stability of therapeutic proteins, and the levels and positions of free cysteines in proteins are closely monitored by both manufacturers and regulators to ensure safety and efficacy. This review summarizes the latest methodologies for the detection and quantification of free cysteines.

## Introduction

Cysteine residues (Cys) in proteins are the most conserved residues throughout the entire proteome. They are redox-active, meaning that they can be oxidized or reduced, and this imparts several distinct functions such as active site catalytic functions in enzymes or forming disulfide bonds (Wong and Hogg, 2010). Disulfide bonds are the covalent bonds formed between the oxidized sulfur atoms of Cys residues and provide mechanical stabilization of protein tertiary and quaternary structures. This is particularly true for proteins that reside extracellularly where disulfide bonds help protect them from the harsh pH-variable, protease-rich environment (Pace et al., 1988).

**FIGURE 1:**
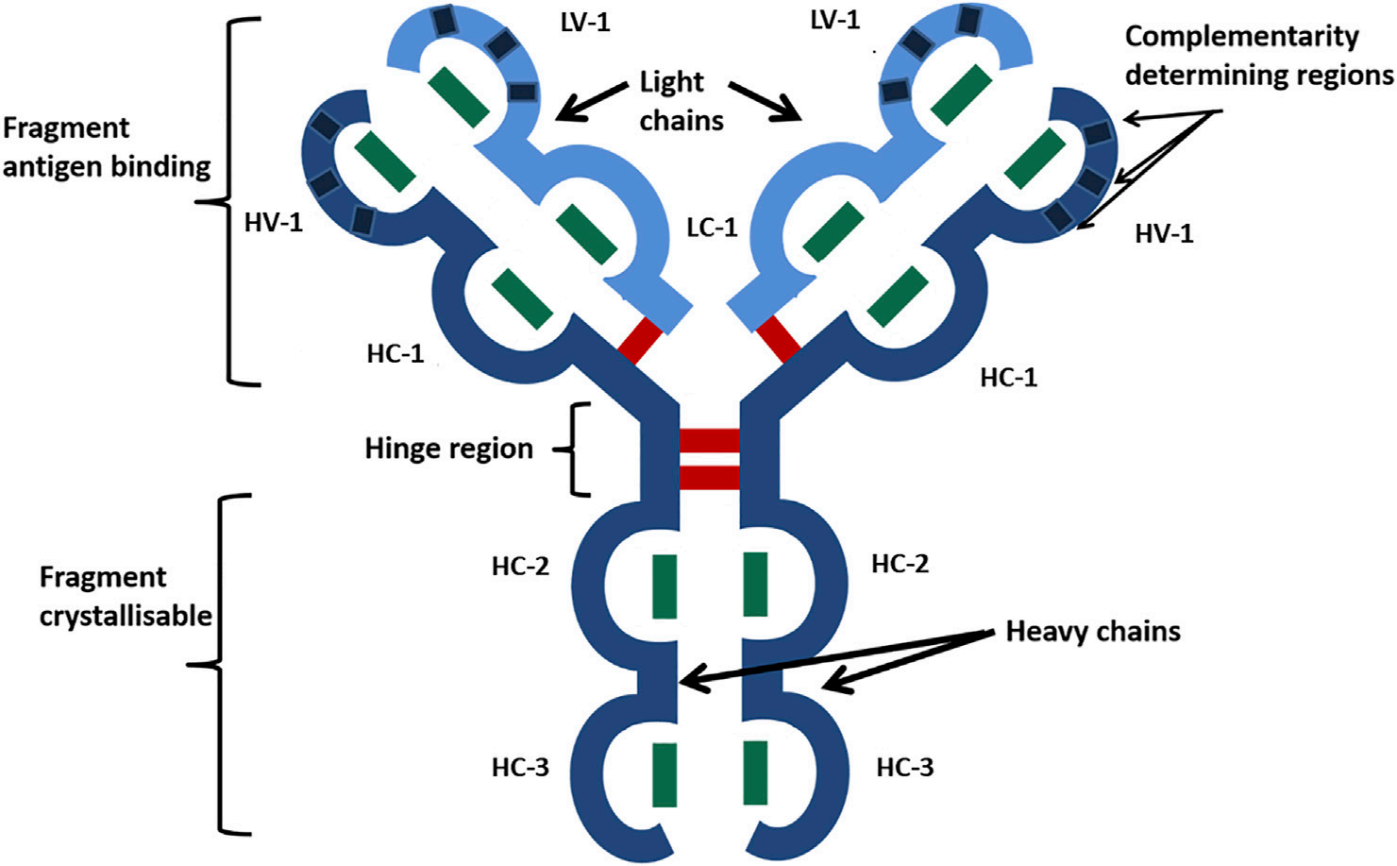
Schematic of the IgG1 mAb characteristic Y-shaped structure. Heavy chains are shown in dark blue, and light chains in are shown light blue. Ig-like domains are represented as bulges in the linear protein sequence and are named HV, heavy variable; HC, heavy constant; LV, light variable; and LC, light constant. Disulfide bonds are represented by bars, with green being disulfide bonds buried within Ig domains and red being exposed interchain disulfide bonds. Reproduced with permission from Gurjar et al., 2019.

The recombinant DNA technology has facilitated the bulk production of biotherapeutic proteins. In particular, immunoglobulins (Ig) have been utilized in the form of monoclonal antibodies (Carrara et al., 2021) (mAbs) to treat many inflammatory diseases and cancers. Immunoglobulin gamma subtype 1 (IgG1) is the most common mAb scaffold in antibody therapeutics (Shepard et al., 2017) and consists of two light chains (composed of two Ig domains each) and two heavy chains (formed from four Ig domains each). Figure 1 shows how these chains are arranged to form the distinctive Y-Shape of IgG1 with each of the Ig domains stabilized by a buried intrachain disulfide bond, with the quaternary structure stabilized by four interchain disulfide bonds, giving 16 in total (Janeway et al., 2001).

Although the disulfide bonding patterns of IgG1 are well conserved and there are relatively few noncanonical Cys found, even in the variable region, free-Cys have been detected in Ig extracted from sera and recombinantly produced mAbs. The majority of detected free-Cys arises from incomplete processing within the host cell during manufacture where high conditions of cellular stress are encountered or through extracellular reduction by intercellular host proteins such as thioredoxin in the harvest and purification of mAbs. Free-Cys arising from disulfide bond reduction in mAbs is undesirable due to the negative effects this has on affinity (Harris, 2005), functions (Gurjar et al., 2019), aggregation (Trivedi et al., 2009; Buchanan et al., 2013; Chung et al., 2017), and stability (Lacy et al., 2008); manufacturers go to great lengths to minimize the amount of free-Cys in therapeutic mAb preparations (Trexler-Schmidt et al., 2010). Although as yet there are no guidelines from regulators on acceptable levels, manufacturers justify the levels on a safety and efficacy basis for each product. Furthermore, the development of structurally diverse next-generation therapeutic antibody platforms and antibody-drug conjugates exogenous cysteines are often added to stabilize structures (Sawant et al., 2020) or to conjugate payloads (You et al., 2021). The methods discussed herein can easily be adapted to quantify the level of free-Cys in these systems.

**FIGURE 2:**
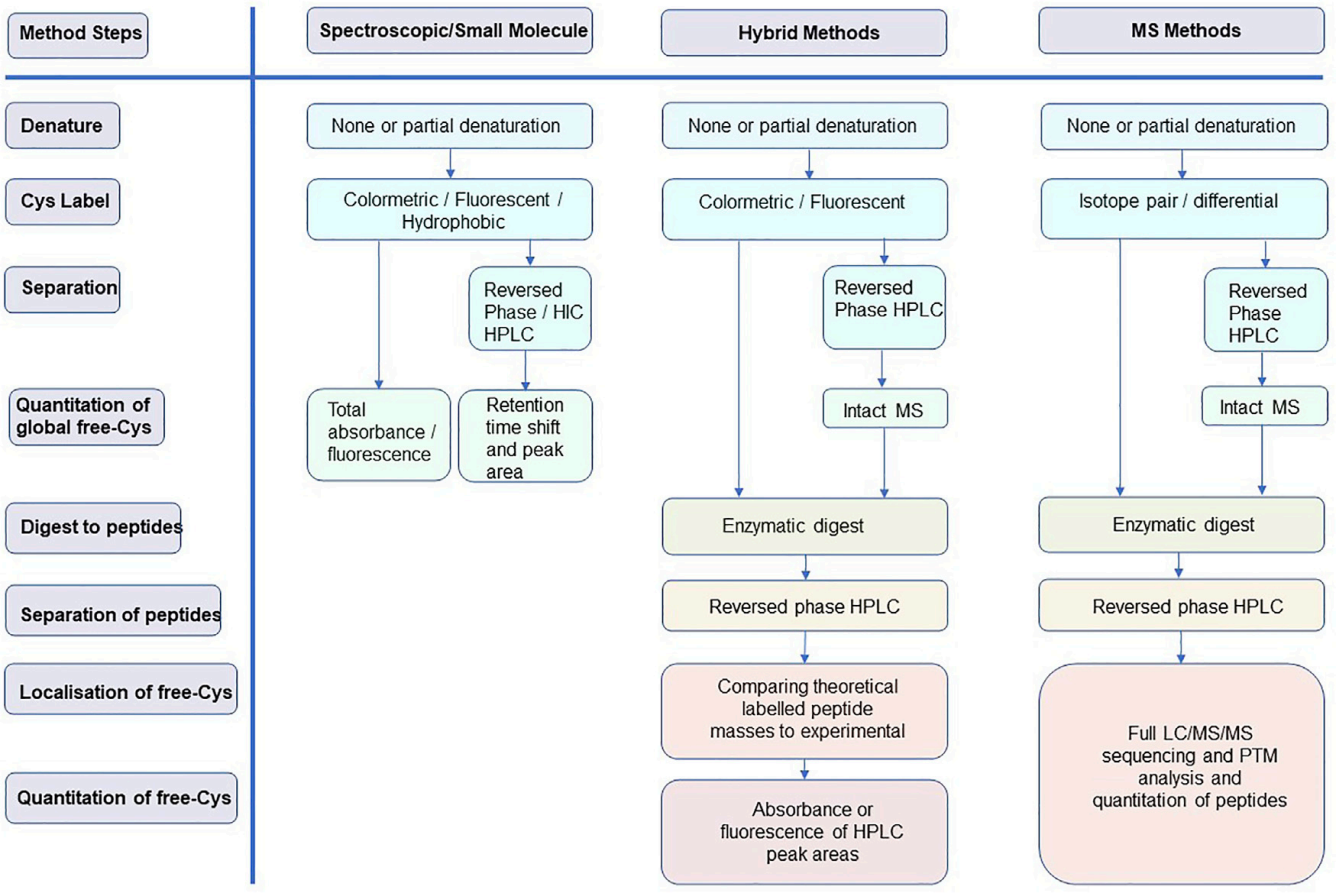
Typical workflows for the three method classes discussed in this review highlighting how each of the method steps is addressed.

The purpose of this study is to review the relevant methods for identifying and quantifying free-Cys in proteins, with a focus on mAbs. Pros and cons are discussed to provide insight into methodologies and inform readers so that they are able to select and improve upon their application. The focus is on methodologies developed over the last 15 years and is presented in three sections of increasing technical complexity: 1) spectroscopic methods, 2) hybrid spectroscopic-mass spectrometry methods, and 3) wholly mass spectrometry-based methods. Typical workflows for each of these method classes are represented in Figure 2.

## Spectroscopic Methods

The first, reliable spectroscopic method for the determination of free-Cys in proteins was developed by Ellman (Ellman, 1959). Free-Cys in a protein are reacted with 5,5-dithio-bis(2-nitrobenzoic acid) (DTNB) forming stable yellow-colored 2-nitro-5-thiobenzoic acid (TNB), which can be quantified by measuring the absorbance at 412 nm and applying a molar extinction coefficient of 13,600 M^−1^ cm^−1^; however, with a limit of detection of around 3 μM free-Cys, the method is not sensitive enough for the detection of the low levels encountered in mAbs. Wright et al. (Wright and Viola, 1998) used a systematic approach to greatly increase the sensitivity of DNTB down to 0.3 µM using carefully controlled conditions such as dialysis, an extended range of standards, and careful control of protein to reagent ratios with accurate quantitation of protein concentration. Partial denaturing conditions and control of reaction times can be used to identify surface accessible versus buried Cys. They also utilized the fluorescent reagent monobromobimane (mBBr), which reacts with free-Cys to form fluorescent adducts which emit at 360 nm when excited at 280 nm. Free-Cys are routinely detectable down to 1 μM, and concentrations as low as 10 nM can be achieved using high-performance liquid chromatography (HPLC) separation of the labeled proteins with the Cys adducts detected using a spectroscopic detector (Fahey et al., 1981). ThioGlo reagents are naphthopyranones derivatized with maleimide which react rapidly with accessible Cys and produce a highly fluorescent product (Langmuir et al., 1995) with sensitivity down to 50 fmol and much increased reproducibility with HPLC methods (Ercal et al., 2001).

An interesting take on colorimetric quantification of thiols is the papain amplification assay (Singh et al., 1993; Singh et al., 1995), in which an inactivated mixed disulfide form of papain is reacted with a thiol to generate a stoichiometric amount of papain. This is assayed using an N-benzoyl-l-arginine-p-nitroaniline substrate that releases the chromogenic substrate p-nitroaniline. As this is an amplification assay, very high levels of sensitivity approaching those of fluorescence can be reached.

Although none of the aforementioned methods have been specifically applied to free-Cys in mAbs, there is no reason why they could not be adapted to perform quick, reliable quantitation of therapeutic mAbs. However, in 2002, Zhang et al. undertook a detailed study on quantifying free-Cys in mAbs using N-(1-pyrenyl) maleimide, which fluoresces at 380 nm when covalently linked to a Cys (Zhang and Czupryn, 2002). To quantify, a standard curve of NPM-derivatized N-acetyl-Cys was used and free-Cys levels of 0.02 and 0.1 mol/mol of protein under native and denaturing conditions, respectively, were detected.

An interesting development of maleimide labeling has been recently employed, where free-Cys in mAbs are labeled with N-tert-butylmaleimide (NtBM) (Welch et al., 2018). When unlabeled and NtBM-labeled mAb are analyzed on C4 RP-HPLC, there is a retention time shift associated with NtBM-labeled mAb, allowing resolution of the two peaks and quantitation of free-Cys. A similar method utilizes N-cyclo-hexylmaleimide as the free thiol label and hydrophobic interaction chromatography for separation (Wei et al., 2019).

## Hybrid Spectroscopic/Mass Spectrometry Methods

Combining mass spectrometry with spectroscopic quantitation of free-Cys, Chumsae et al. (2009) reported a combined fluorescent label and mass spectrometry approach that not only identifies the number of free-Cys but also simultaneously localizes them. Five recombinant IgG1 mAbs were first treated with 5-iodoacetamidofluorescein (5-IAF) under partially denaturing conditions of 4M guanidine hydrochloride to expose all free sulfhydryl groups; a 10:1 5-IAF:mAb ratio ensured efficient alkylation and ‘fixed’ the redox state of free-Cys. Following this, remaining disulfide-bonded Cys were differentiated from the nonbonded Cys by reacting them with IAA after full denaturing and reduction. Initial analysis by matrix-associated laser desorption/ionization time of flight mass spectrometry (MALDI-Tof MS) revealed the quantified number of free-Cys as each one alkylated with 5-IAF gave a mass shift of 387.4 Da. Then, to determine *where* the free-Cys resided, mAbs were digested with trypsin and tryptic peptides separated by RP-HPLC using fluorescence detection at 520 nm specific for 5-IAF-modified peptides. These peaks were collected and the sequences were determined by MALDI-Tof MS, allowing the determination of the positions and domain localization of the free cysteines.

Huh et al. (2013) used a similar approach but with a fluorescent Alexa Fluor C-5-coupled maleimide reagent (AF594) as the probe for free-Cys in several recombinant IgG1 and IgG2 mAbs. Under partial denaturing conditions (7M guanidine HCl), the intact antibodies showed similar levels of free-Cys using total AF594 fluorescence and RP-HPLC separation to that of DNTB; mAb constant domains contain 1–2.7% free-Cys for IgG1 and 1–2.8% for IgG2. To identify the free-Cys-containing peptides, the mAbs were Lys-C-digested and separated on RP-HPLC, and the experimental masses of the peptides plus labels compared to theoretical masses. Comparison of the total fluorescence at 594 nm of the peptide peaks to an AF594 standard curve was used to quantitate the levels of free-Cys per peptide. They then applied these methods to show that mechanical agitation of the mAbs results in breakage of disulfide bonds and covalent aggregation via the liberated Cys.

## Wholly Mass Spectrometry Methods

Coupling stable isotope pairs to differentially alkylate free-Cys to high sensitivity, high-resolution LC-MS/MS provides the most comprehensive analysis of the redox and disulfide-bonded state of Cys in proteins/mAbs. Xiang et al. used ^12^C-iodoacetic acid (^12^C-IAA) and ^13^C-iodoacetic acid (^13^C-IAA) to differentially label five mAbs and quantify the levels and location of free-Cys in the mAb sequence. The 2 Da mass shift between the labels meant that the authors could identify, and distinguish between, the free Cys originally present in the mAbs and the free Cys liberated from denaturation and reduction of the mAbs. Liquid chromatography–mass spectrometry (LC-MS) was performed after multi-enzyme digest (trypsin, Lys-C, chymotrypsin, Asp-N, or Glu-C), and MS peaks for each peptide were identified from calculated masses of the peptide sequence plus any Cys modification. They calculated peptide isotope peak areas from MS^1^ spectra for both the ^12^C-IAA and ^13^C-IAA peptide adducts to get relative percentages of each form. Spiking experiments showed they could accurately quantify down to 0.5% free-Cys for each peptide, and over the five mAbs they studied, they found levels of free-Cys ranging from 1.5 to 5.6%, with the heavy chain CH3 domain having the highest level of free-Cys. The same group went on to further utilize this method in determining the stability of each disulfide bond in IgG1 mAbs (Liu et al., 2010). It should be noted that the main pitfall of this method is that it is limited to the LC-MS analysis and does not make use of modern LC-MS/MS peptide sequencing and modification localization, therefore relying on time-consuming manual matching of LC-MS peaks with theoretical predicted peptide masses.

A similar method that did use a full nano-LC-MS/MS analysis used ^18^O^−^-labeled iodoacetamide (^18^O-IAA), whereby differential labeling was carried out by alkylating free-Cys with normal IAA, denaturing, reducing, and alkylating with ^18^O-IAA, trypsin digestion, and LC-MS/MS analysis (Wang and Kaltashov, 2012; Wang and Kaltashov, 2015). The percentage free-Cys was calculated using the peak area of the extracted ion chromatogram for a given ^18^O-IAA-labeled peptide from the total ion chromatogram and quoted as a percentage of the total area of IAA + ^18^O-IAA-extracted ion chromatograms. Recombinant human transferrin was used as a model protein, but this could easily be applied to mAbs or other therapeutic proteins.

In another example reported by Chiu (Chiu, 2019), a stable isotope pair of 2-iodo-N-phenylacetamide (^12^C-IPA) and its carbon-13 derivative (^13^C-IPA) was used. This pair has a 6 Da mass difference, and the hydrophobic nature of the alkylating agent allows it to penetrate the hydrophobic core of protein domains without the need for partial denaturation, as demonstrated by quantitation of disulfide bond redox states in the platelet integrin αIIb βIII (Chiu, 2019; Pijning et al., 2021) and Influenza A Hemagglutinin (Florido et al., 2021). Differential alkylation, data analysis, and determination of % free-Cys were performed as in the aforementioned method.

In addition, stable isotopes of iodoacetamide Cys alkylating agents here are also stable isotopes of maleimide-derived Cys alkylating agents. N-Ethylmaleimide (d_0_-NEM) and d^5^-N-ethylmaleimide (d_5_-NEM) can be used differentially to alkylate cysteines within a protein with the d_5_-NEM producing a 5 Da mass shift compared to d_0_-NEM. An early example of this was the quantification of thioredoxin-catalyzed disulfide bond reduction in the cell surface receptor CD44 (Kellett-Clarke et al., 2015). This maleimide chemistry was also used by Robotham et al., who devised a complete strategy for the quantitation of free-Cys in mAbs at both the intact mAb level and the peptide level (Robotham and Kelly, 2019). They looked at three commercially available mAbs and used B-lactoglobulin A as a control as it is known to have a free-Cys. These proteins were reacted with maleimide-PEG_2_-Biotin (MPB), which adds a mass of 525 Da per labeled Cys, and the intact labeled mAbs were analyzed by LC-MS. In spiking experiments, the authors could quantify mAbs containing a free-Cys down to less than 2% (∼0.02 mol SH per mol protein) of the total mAb population. Furthermore, reduction of the labeled mAbs with TCEP allowed the independent analysis of the heavy and light chains; thus, the percentage of MPB labeling on each of the heavy and light chains could be ascertained. All their estimations of free-Cys levels agreed well with spectroscopic methods. They went on to demonstrate site-specific quantitation of free-Cys in different redox states by differentially labeling using the d_0_-NEM/d_5_-NEM isotope pair. mAbs partially reduced by 6M guanidine hydrochloride were labeled at pH 5.5 with d_0_-NEM, after which the mAbs were fully denatured and reduced prior to further labeling with d_5_-NEM. The mAbs were deglycosylated with PNGase, trypsin-digested, and subjected to LC-MS/MS analysis. Of the 17 cysteine residues in SigmaMAb, 16 were identified and the percentage of free-Cys was calculated by comparing the area of d_0_-NEM-labeled peptide to the total area of d_0_-NEM + d_5_-NEM-labeled peptides. Again, spiking experiments showed that <2% free-Cys per peptide could be easily detected.

More recently, we developed a differential alkylation strategy to investigate mAbs that does not require stable isotope pairs (Gurjar et al., 2019). Instead, IAA is used to initially alkylate native free-Cys, and then NEM is subsequently used to alkylate free-Cys liberated upon denaturation and reduction. Additionally, a “standard” is prepared—in this case, an mAb fully denatured, reduced, and 100% alkylated with IAA. A label-free LC-MS/MS analysis is performed to sequence the peptides and localize the alkylated Cys; then, extracted peak areas of IAA-labelled Cys peptides in the sample runs are compared to extracted peak areas of the same IAA-labeled Cys peptide in the 100% standard (control). Non-Cys-containing peptides are used to normalize the intensities of the peptides between the different LC-MS/MS runs. The method was applied to five therapeutic mAbs, allowing quantitation of the redox state and amount of free-Cys after various treatments, with sensitivity down to 2%. This has since been successfully applied to determine free-Cys levels in therapeutic recombinant coagulation factor VIII products (Arsiccio et al., 2022). A recent development of this method forgoes the use of a fully NEM-alkylated mAb standard and directly quantified NEM-labeled free-Cys with IAA-labeled Cys derived from disulfide bonds (Li et al., 2021). However, it should be noted that the ionization properties of NEM- and IAA-labeled peptides will not be comparable and may potentially lead to inaccurate quantitation which should be addressed in detailed method validation.

## Discussion

Since the 1959 study by Ellman (Ellman, 1959), the sensitivity and limits of free-Cys quantification in proteins have steadily increased as technologies have evolved. Pure spectroscopic methods permit the quantitation of free-Cys within a protein/mAb but do not inform on where the free-Cys resides. A hybrid approach combining spectroscopic free-Cys detection with mass spectrometry can provide partial localization information but no detailed information. This is because it only utilizes MS to provide an experimental peptide mass that is then compared to the theoretical mass of the peptide in question plus any additional probe mass—no peptide sequencing occurs. As such, it requires a lot of manual annotation and an offline data analysis.

The evolution of MS/MS peptide sequencing when coupled to the nano-ultra-HPLC separation technology allows for the fast and efficient sequencing of peptides along with identification and quantitation of any post-translational modification of amino acids either during synthesis or with exogenously added chemical probes (Prus et al., 2019). Furthermore, the use of stable isotopes of the same thiol-reactive probe allows for the quantitation of free-Cys levels in a single mass spectrometry run, alleviating the need for any standard curves to be generated and therefore any commutability issues that may occur between the behavior of the standards and the samples to be analyzed. Isotope pairs of Cys-reactive probes offer a means of identifying which label has alkylated the Cys on a given peptide based on a mass shift between the heavy and light probes; however, they possess the same physicochemical characteristics showing no difference in specificity or reactivity, nor do they introduce different ionization characteristics or retention time shifts into the LC-MS/MS analysis. Despite this, the stable isotope technology has mainly evolved in the redox-labile allosteric disulfide bond field where it is utilized in determining the relative reactivity of multiple disulfide bonds within a protein as well as the percentage of reduction in each disulfide bond, either in the native state or after treatment (Cook and Hogg, 2013). Strangely, given its ease of use and sensitivity, its uptake in quantifying free-Cys in therapeutic proteins such as mAbs has been slow and sparse.

The differing sensitivity and complexity of the methods mean that varying amounts of protein are needed for each analysis. This ranges from low mg to high μg for spectroscopic methods, especially if coupled with HPLC, to low μg and below for the LC-MS/MS methods. Both methods lend themselves to analysis at different stages of recombinant mAb development and manufacture. For example, if the desire is to monitor the overall level of free-Cys in an mAb product at different manufacturing stages, online spectroscopic methods will provide a good inline scalable solution. However, at the research and development stage, where many clones are being assessed for their stability and the material is at a premium, a full LC-MS/MS analysis might be beneficial to pinpoint the areas of the mAb where the free-Cys is occurring.

## References

[B1] Arsiccio A. Metcalfe C. Pisano R. Raut S. Coxon C. (2022). A Proximity-Based In Silico Approach to Identify Redox-Labile Disulfide Bonds: The Example of FVIII. PLoS One 17, e0262409. 10.1371/journal.pone.0262409 35130281 PMC8820644

[B2] Buchanan A. Clementel V. Woods R. Harn N. Bowen M. A. Mo W. (2013). Engineering a Therapeutic IgG Molecule to Address Cysteinylation, Aggregation and Enhance Thermal Stability and Expression. MAbs 5, 255–262. 10.4161/mabs.23392 23412563 PMC3893235

[B3] Carrara S. C. Ulitzka M. Grzeschik J. Kornmann H. Hock B. Kolmar H. (2021). From Cell Line Development to the Formulated Drug Product: The Art of Manufacturing Therapeutic Monoclonal Antibodies. Int. J. Pharm. 594, 120164. 10.1016/j.ijpharm.2020.120164 33309833

[B4] Chiu J. (2019). Quantification of the Redox State of Protein Disulphide Bonds. Methods Mol. Biol. 1967, 45–63. 10.1007/978-1-4939-9187-7_4 31069764

[B5] Chumsae C. Gaza-Bulseco G. Liu H. (2009). Identification and Localization of Unpaired Cysteine Residues in Monoclonal Antibodies by Fluorescence Labeling and Mass Spectrometry. Anal. Chem. 81, 6449–6457. 10.1021/ac900815z 19572546

[B6] Chung W. K. Russell B. Yang Y. Handlogten M. Hudak S. Cao M. (2017). Effects of Antibody Disulfide Bond Reduction on Purification Process Performance and Final Drug Substance Stability. Biotechnol. Bioeng. 114, 1264–1274. 10.1002/bit.26265 28186329 PMC5413809

[B7] Cook K. M. Hogg P. J. (2013). Post-translational Control of Protein Function by Disulfide Bond Cleavage. Antioxidants Redox Signal. 18, 1987–2015. 10.1089/ars.2012.4807 23198756

[B8] Ellman G. L. (1959). Tissue Sulfhydryl Groups. Archives Biochem. Biophysics 82, 70–77. 10.1016/0003-9861(59)90090-6 13650640

[B9] Ercal N. Yang P. Aykin N. (2001). Determination of Biological Thiols by High-Performance Liquid Chromatography Following Derivatization by ThioGlo Maleimide Reagents. J. Chromatogr. B Biomed. Sci. Appl. 753, 287–292. 10.1016/s0378-4347(00)00560-0 11334342

[B10] Fahey R. C. Newton G. L. Dorian R. Kosower E. M. (1981). Analysis of Biological Thiols: Quantitative Determination of Thiols at the Picomole Level Based upon Derivatization with Monobromobimanes and Separation by Cation-Exchange Chromatography. Anal. Biochem. 111, 357–365. 10.1016/0003-2697(81)90573-x 7247030

[B11] Flórido M. Chiu J. Hogg P. J. (2021). Influenza A Virus Hemagglutinin Is Produced in Different Disulfide-Bonded States. Antioxidants Redox Signal. 35, 1081–1092. 10.1089/ars.2021.0033 33985344

[B12] Gurjar S. A. Wheeler J. X. Wadhwa M. Thorpe R. Kimber I. Derrick J. P. (2019). The Impact of Thioredoxin Reduction of Allosteric Disulfide Bonds on the Therapeutic Potential of Monoclonal Antibodies. J. Biol. Chem. 294, 19616–19634. 10.1074/jbc.ra119.010637 31727737 PMC6926469

[B13] Harris R. J. (2005). Heterogeneity of Recombinant Antibodies: Linking Structure to Function. in State of the Art Analytical Methods for the Characterization of Biological Products and Assessment of Comparability. Basel, Karger: Dev BioI (Basel). Editors Mire-Sluis A. R., 22, 117–127. 16375256

[B14] Huh J. H. White A. J. Brych S. R. Franey H. Matsumura M. (2013). The Identification of Free Cysteine Residues within Antibodies a Potential Role for Free Cysteine Residues in Covalent Aggregation Because of Agitation Stress. J. Pharm. Sci. 102, 1701–1711. 10.1002/jps.23505 23559428

[B15] Janeway C. A. Jr Travers P. Walport M. Shlomchik M. J. (2001)The Structure of a Typical Antibody Molecule. Immunobiology: The Immune System in Health and Disease. 5th edition. New York: Garland Science

[B16] Kellett-Clarke H. Stegmann M. Barclay A. N. Metcalfe C. (2015). CD44 Binding to Hyaluronic Acid Is Redox Regulated by a Labile Disulfide Bond in the Hyaluronic Acid Binding Site. PLoS One 10, e0138137. 10.1371/journal.pone.0138137 26379032 PMC4574955

[B17] Lacy E. R. Baker M. Brigham-Burke M. (2008). Free Sulfhydryl Measurement as an Indicator of Antibody Stability. Anal. Biochem. 382, 66–68. 10.1016/j.ab.2008.07.016 18675772

[B18] Langmuir M. E. Yang J.-R. Moussa A. M. Laura R. Lecompte K. A. (1995). New Naphthopyranone Based Fluorescent Thiol Probes. Tetrahedron Lett. 36, 3989–3992. 10.1016/0040-4039(95)00695-9

[B19] Li X. Xiao L. Kochert B. Donnelly D. P. Gao X. Richardson D. (2021). Extended Characterization of Unpaired Cysteines in an IgG1 Monoclonal Antibody by LC-MS Analysis. Anal. Biochem. 622, 114172. 10.1016/j.ab.2021.114172 33766578

[B20] Liu H. Chumsae C. Gaza-Bulseco G. Goedken E. R. (2010). Domain-level Stability of an Antibody Monitored by Reduction, Differential Alkylation, and Mass Spectrometry Analysis. Anal. Biochem. 400, 244–250. 10.1016/j.ab.2010.02.004 20152794

[B21] Pace C. N. Grimsley G. R. Thomson J. A. Barnett B. J. (1988). Conformational Stability and Activity of Ribonuclease T1 with Zero, One, and Two Intact Disulfide Bonds. J. Biol. Chem. 263, 11820–11825. 10.1016/s0021-9258(18)37859-1 2457027

[B22] Pijning A. E. Blyth M. T. Coote M. L. Passam F. Chiu J. Hogg P. J. (2021). An Alternate Covalent Form of Platelet αIIbβ3 Integrin that Resides in Focal Adhesions and Has Altered Function. Blood 138, 1359–1372. 10.1182/blood.2021012441 34375384 PMC8532129

[B23] Prus G. Hoegl A. Weinert B. T. Choudhary C. (2019). Analysis and Interpretation of Protein Post-Translational Modification Site Stoichiometry. Trends Biochem. Sci. 44, 943–960. 10.1016/j.tibs.2019.06.003 31296352

[B24] Robotham A. C. Kelly J. F. (2019). Detection and Quantification of Free Sulfhydryls in Monoclonal Antibodies Using Maleimide Labeling and Mass Spectrometry. MAbs 11, 757–766. 10.1080/19420862.2019.1595307 30894096 PMC6601545

[B25] Sawant M. S. Streu C. N. Wu L. Tessier P. M. (2020). Toward Drug-like Multispecific Antibodies by Design. Int. J. Mol. Sci. 21, 7496. 10.3390/ijms21207496 PMC7589779 33053650

[B26] Shepard H. M. Phillips G. L. Thanos C. D. Feldmann M. (2017). Developments in Therapy with Monoclonal Antibodies and Related Proteins. Clin. Med. 17, 220–232. 10.7861/clinmedicine.17-3-220 PMC6297577 28572223

[B27] Singh R. Blättler W. A. Collinson A. R. (1995). [20] Assay for Thiols Based on Reactivation of Papain. Methods Enzymol. 251, 229–237. 10.1016/0076-6879(95)51125-3 7651201

[B28] Singh R. Blattler W. A. Collinson A. R. (1993). An Amplified Assay for Thiols Based on Reactivation of Papain. Anal. Biochem. 213, 49–56. 10.1006/abio.1993.1384 8238881

[B29] Trexler-Schmidt M. Sargis S. Chiu J. Sze-Khoo S. Mun M. Kao Y. H. (2010). Identification and Prevention of Antibody Disulfide Bond Reduction during Cell Culture Manufacturing. Biotechnol. Bioeng. 106, 452–461. 10.1002/bit.22699 20178122

[B30] Trivedi M. Laurence J. Siahaan T. (2009). The Role of Thiols and Disulfides on Protein Stability. Curr. Protein Pept. Sci. 10, 614–625. 10.2174/138920309789630534 19538140 PMC3319691

[B31] Wang S. Kaltashov I. A. (2012). A New Strategy of Using O18-Labeled Iodoacetic Acid for Mass Spectrometry-Based Protein Quantitation. J. Am. Soc. Mass Spectrom. 23, 1293–1297. 10.1007/s13361-012-0396-9 22562395 PMC5809132

[B32] Wang S. Kaltashov I. A. (2015). Identification of Reduction-Susceptible Disulfide Bonds in Transferrin by Differential Alkylation Using O16/O18 Labeled Iodoacetic Acid. J. Am. Soc. Mass Spectrom. 26, 800–807. 10.1007/s13361-015-1082-5 25716754 PMC4401651

[B33] Wei B. Han G. Tang J. Sandoval W. Zhang Y. T. (2019). Native Hydrophobic Interaction Chromatography Hyphenated to Mass Spectrometry for Characterization of Monoclonal Antibody Minor Variants. Anal. Chem. 91, 15360–15364. 10.1021/acs.analchem.9b04467 31747256

[B34] Welch L. Dong X. Hewitt D. Irwin M. Mccarty L. Tsai C. (2018). Facile Quantitation of Free Thiols in a Recombinant Monoclonal Antibody by Reversed-phase High Performance Liquid Chromatography with Hydrophobicity-Tailored Thiol Derivatization. J. Chromatogr. B 1092, 158–167. 10.1016/j.jchromb.2018.05.039 29906677

[B35] Wong J. W. H. Hogg P. J. (2010). Analysis of Disulfide Bonds in Protein Structures. J. Thromb. Haemost. 8, 2345. 10.1111/j.1538-7836.2010.03894.x 20456749

[B36] Wright S. K. Viola R. E. (1998). Evaluation of Methods for the Quantitation of Cysteines in Proteins. Anal. Biochem. 265, 8–14. 10.1006/abio.1998.2858 9866701

[B37] You J. Zhang J. Wang J. Jin M. (2021). Cysteine-Based Coupling: Challenges and Solutions. Bioconjugate Chem. 32, 1525–1534. 10.1021/acs.bioconjchem.1c00213 34105345

[B38] Zhang W. Czupryn M. J. (2002). Free Sulfhydryl in Recombinant Monoclonal Antibodies. Biotechnol. Prog. 18, 509–513. 10.1021/bp025511z 12052067

